# Efficiency of metaphase II oocytes following minimal/mild ovarian stimulation in vitro fertilization

**DOI:** 10.1186/s40738-016-0025-6

**Published:** 2016-09-01

**Authors:** John J. Zhang, Mingxue Yang, Zaher Merhi

**Affiliations:** 1 Reproductive Endocrinology and Infertility, New Hope Fertility Center, New York, NY USA; 2 Division of Reproductive Biology, Department of Obstetrics and Gynecology, New York University School of Medicine, 180 Varick Street, sixth floor, New York, NY 10014 USA

**Keywords:** IVF, Minimal ovarian stimulation, Mild ovarian stimulation, MII oocyte, Live birth

## Abstract

**Background:**

An inverse relationship between oocyte efficiency and ovarian response was reported in conventional IVF. The purpose of this study was to report metaphase II (MII) oocyte efficiency according to oocyte yield in minimal/mild stimulation IVF (mIVF) and to assess whether oocyte yield affects live birth rate (LBR).

**Methods:**

Infertile women (*n* = 264) aged < 39 years old with normal ovarian reserve who had mIVF were recruited. All participants received the same protocol for ovarian stimulation. All the embryos were cultured to the blastocyst stage and vitrified using a freeze-all approach. This was followed by a single blastocyst transferred to each participant in subsequent cycles over a 6-month period. Ovarian response was categorized according to the number of MII oocyte yield (low: 1–2, intermediate: 3–6 and high ≥ 7 MII oocytes). MII oocyte utilization rate was calculated as the number of live births divided by the number of MII oocytes produced after only one oocyte retrieval and subsequent transfers of vitrified/warmed blastocysts. The main outcome measure was cumulative LBR over a 6-month period.

**Results:**

Among all the participants, 1173 total retrieved oocytes (4.4 ± 0.2 per patient) resulted in 1019 (3.9 ± 0.2 per patient) total MII oocytes, a clinical pregnancy rate of 48.1 % and a LBR of 41.2 %. Oocyte utilization rate was inversely related to ovarian response where it was 30.3 % in the “low” vs. 9.3 % in the “intermediate” vs. 4.3 % in the “high” oocyte yield groups (*p* < 0.05). Implantation rate significantly dropped as the number of MII oocytes increased and was highest in the “low” oocyte yield group (*p* < 0.0001). Cumulative LBR was similar in “low,” “intermediate,” and “high” oocyte yield groups (*p* > 0.05). The number of MII oocytes had poor sensitivity and specificity for predicting a live birth.

**Conclusion:**

These data extend the hypothesis of oocyte efficiency reported in conventional IVF protocols to mIVF protocols.

**Trial registration:**

Registration clinicaltrials.gov: NCT00799929.

## Background

In the late 1980s and early 1990s, the widespread use of high doses of gonadotropins was introduced in in vitro fertilization (IVF) protocols [1, 2]. Conventional IVF using high doses of gonadotropins has several advantages that include the retrieval of high number of oocytes and the formation of high numbers of embryos, leading to higher pregnancy rates [1, 2]. Despite these improved success rates, conventional ovarian stimulation IVF has several drawbacks such as high cost and ovarian hyperstimulation syndrome (OHSS). Due to these drawbacks, more physicians are revisiting and resorting to minimal/mild ovarian stimulation IVF (mIVF) protocols which usually yield a low number of oocytes [3–7]. Historically however, a low number of oocytes after follicular aspiration has been associated with diminished pregnancy outcomes, often attributed to ovarian aging [8]. On the other hand, the association between low yield of oocytes in young women with normal ovarian reserve and pregnancy outcomes has been understudied.

Minimal and mild ovarian stimulation IVF usually refers to the use of low-dose gonadotropins with or without a sequential administration of clomiphene citrate [4–7]. Minimal/mild ovarian stimulation usually yields a maximum of five to six oocytes [5]. A relatively low number of oocytes retrieved after mild ovarian stimulation distinctly differs from the pathological reduction in the number of oocytes retrieved after profound ovarian stimulation (poor ovarian response) which is associated with poor IVF outcome, such as in women with diminished ovarian reserve [9]. Notably, it has been suggested that the relatively small number of oocytes obtained after mild ovarian stimulation may represent the best of the cohort in a given cycle since a study of mild stimulation protocols reported that cycles with four or fewer retrieved oocytes resulted in a 67 % pregnancy rate in good responders [9]. Interestingly, a study demonstrated that cumulative live birth rate (LBR) and patients' discomfort (for example, pain at the injection site) were similar in patients who had mild ovarian stimulation followed by a single embryo transferred compared to those who had conventional IVF followed by two embryos transferred [10]. However, data pertaining to the efficency, in particular live birth, of mature oocytes produced by mIVF in normal responders is understudied. Additionally, data pertaining to the assessment of reproductive efficiency based on oocyte yield of human oocytes fertilized in vitro is scarse [11]. In conventional IVF cycles, it has been reported that the oocyte utilization rate in women aged ≤ 37 years was approximately 5 % live birth per mature oocyte [11]. The purpose of this study was to report metaphase II (MII) oocyte efficiency following mIVF with cumulative LBR as the main outcome.

## Methods

### Participants

Between February 2009 and August 2013, 285 participants were allocated to mIVF and freeze-all embryo strategy, followed by transfers of single frozen/thawed blastocysts. Women aged between 18 and 38 with normal menstrual cycles and requiring a first IVF cycle treatment were included. Inclusion criteria included women with infertility diagnosis of unexplained, male and tubal factors. Women with pre-existing medical conditions and those with body mass index (BMI) <18.5 or >32 kg/m^2^ were excluded. Additionally, women who had a cycle day 3 follicle stimulating hormone (FSH) ≥ 13 mIU/mL were excluded for possible diminished ovarian reserve. The study was approved by the Institutional Review Board of New York Downtown Hospital (IRB approval reference number: JZ-09-08). The study protocol was approved by the institutional review boards of the New York Downtown Hospital and the Biomedical Research Alliance of New York (BRANY). Informed consent was obtained from each participant before recruitment. This study was a secondary analysis of data from a randomized controlled trial published elsewhere [12].

### mIVF protocol using freeze-all embryo strategy

Each participant had only one ovarian stimulation and one oocyte retrieval. All formed blastocysts were vitrified then they were transferred as one-by-one in subsequent frozen embryo transfer cycles (i.e., cycle after cycle without any break between cycles). Thus, each participant had a single embryo transfer at the blastocyst stage.

All participants had similar ovarian stimulation protocol. After oral contraceptive pill pre-treatment for approximately 3 weeks and adequate suppression, minimal/mild ovarian stimulation was started with an extended regimen (from cycle day 3 until the day before triggering) of clomiphene citrate (50 mg/day orally) in conjunction with low dose of gonadotropin (75 IU’s daily) injections (Bravelle and/or Menopur, Ferring, Parsippany, NJ; Follistim, Merck, White House Station, NJ; or Gonal F, EMD Serono, Rockland, MA) starting on cycle day 4–7. No hypothalamic-pituitary suppression using gonadotropin releasing hormone (GnRH) agonist or antagonist was used. The final maturation of oocytes was induced by a nasal GnRH agonist (Synarel nasal spray 2 mg/mL, Pfizer, New York, NY) when the lead follicle was > 18 mm. Retrieved oocytes were fertilized by IVF or ICSI as clinically indicated. All embryos were cultured until the blastocyst stage and then vitrified.

### Embryo culture, vitrification, thawing and frozen embryo transfer

All blastocysts that have distinct inner cell mass and trophectoderm were vitrified using the CryoTop method (Kitazato Biopharma) [13]. In brief, Embryos were cultured from day 1 in Astec tri-gas incubator, at 37 °C, 6 % CO_2_, and 5 % O_2_ using Global Total media (LGGT, LifeGlobal), with sterile mineral oil overlay (LGOL, LifeGlobal). The embryos were placed in drops of Equilibration Solution (ES) containing 7.5 % (vol/vol) ethylene glycol (EG) + 7.5 % dimethyl sulfoxide (DMSO) at room temperature. Then, they were transferred into a Vitrification Solution (VS) drop containing 15 % EG + 15 % DMSO + 0.7 M sucrose + Ficoll 0.01 g/ml. Embryos were then placed in ES media for 10 min, and transferred into VS media for 1 min, then loaded onto cryotop (only one embryo per cryotop) and submerged into liquid nitrogen immediately. For embryo thawing, the cryotop was quickly removed from the liquid nitrogen and the tip was submerged in T1 media (1 M Sucrose + Base Solution). The embryo was then placed in T1 for 1 min before being transferred to T2 (0.75 M Sucrose + Base Solution), T3 (0.50 M Sucrose + Base Solution), T4 (0.25 M Sucrose + Base Solution), and T5 (Base Solution) media, for 3 min each. Upon completion of the thaw, the embryo was washed in several drops of ES media.

A single thawed blastocyst was transferred in a subsequent natural or artificially prepared cycle with oral Estrace (Actavis Pharma, Inc, Parsippany, NJ) [14] within a 6-month period from the oocyte retrieval. When available, back-to-back blastocyst transfers were performed in a single participant.

### Statistical analysis

Cycles were subdivided into three subgroups according to the number of mature MII oocyte yield following oocyte retrieval: “low” represented a yield of 1–2 oocytes; “intermediate” represented a yield of 3–6 oocytes; and “high” represented a yield of ≥ 7 oocytes. Because the aim of the study was to evaluate the efficiency of MII oocytes, patients who had no oocytes retrieved were excluded from the data analysis.

The primary outcome was cumulative live birth rate. Secondary outcomes included cumulative implantation rate, cumulative clinical pregnancy rate, number of blastocysts formed, total dose of gonadotropins used per cycle, and number of fertilized oocytes per MII oocytes. The cumulative implantation rate was defined as the number of gestational sacs observed on ultrasound at 6 weeks of pregnancy divided by the number of embryos transferred. A cumulative clinical pregnancy was defined as at least one intrauterine sac at 6 weeks gestation and live birth was defined as a child born after 22 weeks of gestation or weighing at least 500 g. The cumulative clinical pregnancy rate over a 6-month period was calculated as the number of pregnancies divided by the total number of participants. The cumulative live birth rate over a 6-month period was calculated as the number of births divided by the total number of participants. The cumulative live birth was expressed as odds ratio (OR) with corresponding 95 % confidence interval (CI). The mean MII oocytes (3.9 ± 0.2) and the cumulative live birth rate of 41.2 % in all the cohort was used as the reference group for OR calculations. Oocyte utilization rate was calculated as the number of live births *over a 6-month period* divided by number of MII oocytes produced following *only one* ovarian stimulation and *only one* oocyte retrieval. For continuous outcomes, data were expressed as mean ± standard error of the mean (SEM) and *t-*test or ANOVA were used appropriate. Chi-square test was used for categorical data. Multivariate logistic regression analyses were conducted to identify “low,’ “intermediate,” and “high” ovarian responses as independent correlates of a live birth. In order to determine the predictive ability of the number of MII oocytes and age (the strongest predictor of achieving a live birth), receiver operating characteristic (ROC) curves were performed and the area under the ROC curve (AUC) was determined. *P* < 0.05 was considered statistically significant. *GraphPrism* Software was used to perform all statistical analyses.

## Results

There were 264 participants out of 285 who had minimal/mild IVF treatment and had oocytes retrieved. The remaining 21 participants were excluded for reasons such as spontaneous pregnancy, persistent ovarian cysts, premature ovulation and withdrawal from the study participation. The demographics and clinical characteristics of the participants are summarized in Table 1. There was no difference in age or day 3 FSH among the “low”, “intermediate,” or “high” MII oocyte yield groups (32.4 ± 0.4 vs. 32.6 ± 0.3 vs. 31.9 ± 0.6, respectively; *p*-value = 0.6 for age, and 9.0 ± 0.2 vs. 8.5 ± 0.2 vs. 8.3 ± 0.4, respectively; *p*-value = 0.2 for day 3 FSH). However, participants in the “high” MII oocyte yield group had signifincalty higher BMI and received higher doses of gonadotropins (Table 1). There was no difference in cycles requiring ICSI (*p* > 0.05) among the three groups. The 264 participants produced a total of 1173 retrieved oocytes (mean ± SEM = 4.4 ± 0.2 per patient; range: 1–23). Of these oocytes, a total of 1019 (mean ± SEM = 3.9 ± 0.2 per patient, range 1–18) were MII oocytes, yielding a maturity rate of 86.9 %, a cumulative clinical pregnancy rate of 48.1 % and a cumulative live birth rate of 41.2 % over a 6-month period. The majority (86.7 %) of the participants had 6 or less MII oocytes. As seen in Table 2, only 13.2 % of participants in this group had “high” responses (≥7 MII oocytes).

**Table 1:** Demographics and clinical characteristics of the participants according to oocyte yield

	All participants (*n* = 264)	Low (1–2 oocytes) *n* = 93	Intermediate (3–6 oocytes) *n* = 136	High (≥7 oocytes) *n* = 35	*p*-value
Age (years)	32.4 ± 0.2	32.4 ± 0.4	32.6 ± 0.3	31.9 ± 0.6	0.6
BMI (kg/m^2^)	24.6 ± 0.2	21.0 ± 0.1^a^	25.6 ± 0.2^b^	30.6 ± 0.2^c^	<0.0001
Nulliparity	193 (73.1)	74 (79.6)	95 (69.8)	22 (62.9)	0.7
Day 3 FSH (mIU/mL)	8.7 ± 0.1	9.0 ± 0.2	8.5 ± 0.2	8.3 ± 0.4	0.2
Day 3 estradiol	53.9 ± 2.0	50.2 ± 2.2	57.7 ± 3.3	49.1 ± 3.5	0.1
Antral follicle count	13.3 ± 0.5	14.2 ± 1.1	12.6 ± 0.4	13.8 ± 1.0	0.3
Days of stimulation	11.1 ± 0.5	12.8 ± 1.3^a^	10.1 ± 0.1^b^	10.2 ± 0.2	0.03
Total dose of gonadotropins	456.6 ± 8.0	427.6 ± 14.4 ^a^	464.3 ± 11.1	503.6 ± 13.9^b^	0.04

Data are presented as mean ± SEM or n (%)

Statistically significant for a *vs* b *vs* c in the same row

**Table 2:** Cycles outcome according to oocyte yield in women who underwent mIVF

	Low (1–2 oocytes) *n* = 93	Intermediate (3–6 oocytes) *n* = 136	High (≥7 oocytes) *n* = 35	*p*-value
# Mature (MII) oocytes	142	551	326	
# Fertilized oocytes (% fertilization rate)	1.4 ± 0.07 (32.1) ^a^	3.4 ± 0.1 (68.1) ^b^	7.6 ± 0.4 (80.7)^c^	<0.0001
# Blastocysts formed per patient (% blastocyst formation rate)	1.2 ± 0.06 (78.4) ^a^	2.6 ± 0.1 (57.0) ^b^	5.5 ± 0.4 (42.6) ^c^	<0.0001
# Blastocysts vitrified	0 ^a^	50 ^b^	28 ^c^	<0.0001
Total number of blastocysts transferred in each group	73 ^a^	186 ^b^	63 ^c^	0.01
% Transferred blastocysts from all vitrified blastocysts	100 ^a^	68.1 ^b^	80.7 ^b^	<0.0001
Miscarriages	17	48	19	0.8
Cumulative clinical pregnancy rate over 6 month period	48 (51.6)	68 (50.0)	15 (42.8)	0.7
Cumulative live birth rate over 6 month period	43^d^ (46.2)	51^e^ (37.5)	14^f^ (40.0)	0.6

Data are presented as mean ± SEM or *n* (%)

Statistically significant for ^a^ *vs* ^b^ *vs* ^c^ in the same row

^d^ 5 pending ongoing pregnancies, ^e^ 17 pending pregnancies, ^f^ 1 pending pregnancy

ANOVA was performed for continuous variables and chi-square test was used for categorical data

**Fig. 1:**
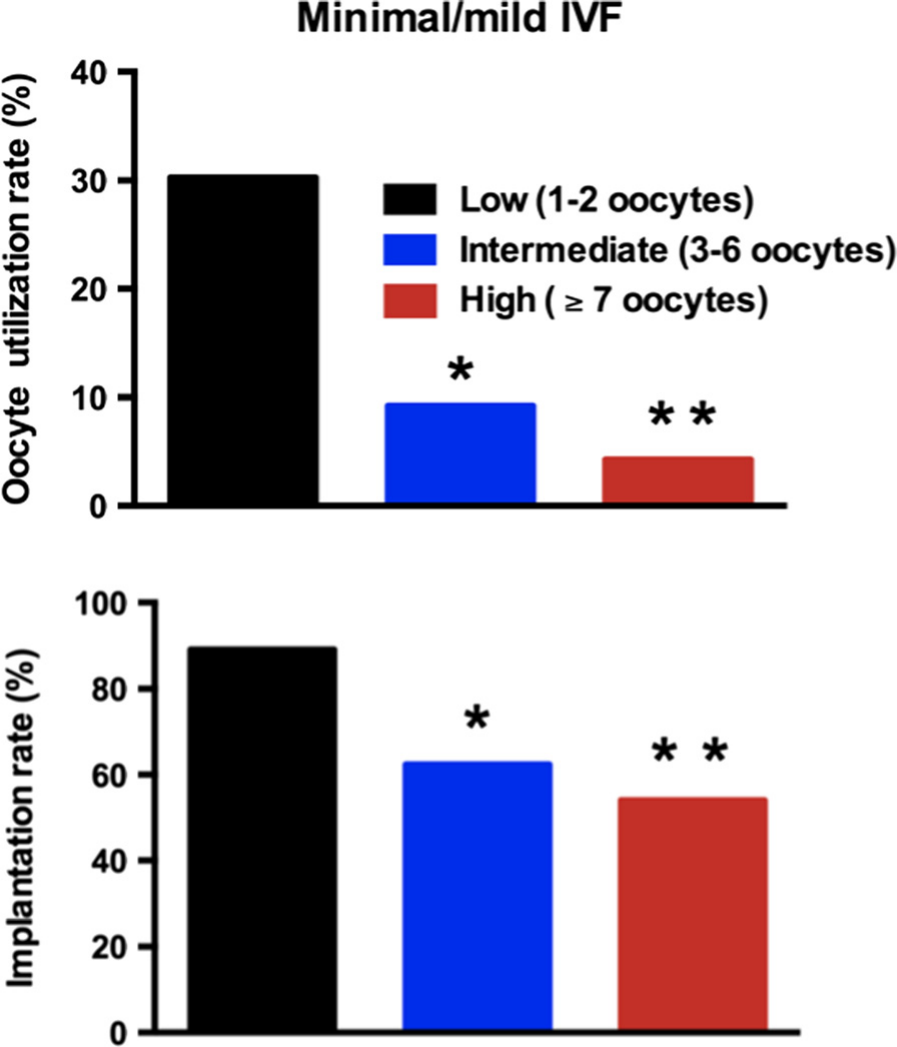
In minimal/mild ovarian stimulation, mature (MII) oocyte utilization rate and cumulative implantation rate declined as the number of MII oocytes increased. *p < 0.05 low vs. intermediate. **p < 0.05 intermediate vs. high

As expected, women in the “high” MII oocyte yield group produced significantly more fertilized oocytes and more blastocysts than women in the “intermediate” MII oocyte yield (*p* < 0.0001, Table 2). Additionally, women in the “intermediate” MII oocyte yield group, in turn, produced significantly more fertilized oocytes and more blastocysts than those in the “low” MII oocyte yield group (*p* < 0.0001, Table 2). There were no frozen blastocysts in the “low” oocyte yield group, but there were 50 frozen blastocysts in the “intermediate” MII oocyte yield group, and 28 frozen blastocysts in the “high” MII oocyte yield group remaining after the 6-month period of the study. Interestingly, cumulative implantation rate was highest in the “low” MII oocyte yield group (89.0 %) followed by the “intermediate” MII oocyte yield group (62.4 %), and it was lowest in the “high” MII oocyte yield group (54.0 %) (*p* < 0.0001; Table 2 and Fig. 1). All participants in the “low,” “intermediate,” and “high” MII oocyte yield groups had single blastocyst transfer (via frozen embryo transfer cycle) and the cumulative clinical pregnancy (*p* = 0.7, Table 2) and cumulative live birth (*p* = 0.6, Table 2) rates did not differ among the three subgroups. MII oocyte utilization rate was inversely related to ovarian response: it was 30.3 % in the “low” *vs* 9.3 % in the “intermediate” *vs* 4.3 % in the “high” MII oocyte yield groups (*p* < 0.05; Fig. 1).

**Fig. 2:**
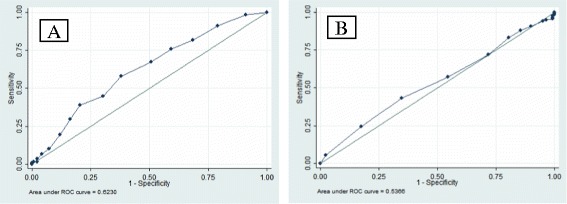
Area (AUC) under receiver operating characteristic (ROC) curves for number of mature (MII) oocytes and age in mIVF. AUC for age (**a**) was statistically significant (*p* = 0.0008) while AUC for number of MII oocytes (**b**) was not (*p* = 0.9)

Among all the participants, logistic regression showed that age was a negative predictor for achieving a live birth (*p* = 0.008). In a multivariate logistic regression adjusting for the number of days of stimulation and total dose of gonadotropins used per cycle, the number of MII oocytes (i.e., “low”, “intermediate,” or “high”) did not affect the likelihood of achieving a live birth (OR = 1.04 [0.96–1.12], *p* = 0.09) among all the participants. Additionally, the yield of MII oocytes according to “low,” “intermediate,” or “high” ovarian response did not affect the likelihood of achieving a live birth in a multivariate logistic regression after adjusting for the number of days of stimulation and total dose of gonadotropins used per cycle (Table 3). ROC analysis was performed in order to determine the predictive ability of age and the number of MII oocytes for live birth as outcome. As shown in Fig. 2, results showed that for the number of MII oocytes, AUC was 0.54 (SE = 0.036, CI = 0.39–0.53; *p* = 0.9). For age, the AUC was 0.62 (SE = 0.032, CI = 0.83–0.95; *p* = 0.0008). The number of MII oocytes had very poor sensitivity and specificity for predicting a live birth.

**Table 3:** Likelihood of live birth according to the yield of MII oocytes obtained in mIVF treatment

MII yield	OR (95 % CI)	*p*-value	Live birth (%)
Low: 1–2 oocytes	0.84 (0.30–2.32)	0.7	46.2
Intermediate: 3–6 oocytes	1.18 (0.83–1.67)	0.3	37.5
High ≥7 oocytes	1.19 (0.97–1.47)	0.06	40.0

The mean MII oocytes (3.9 ± 0.2) and the cumulative live birth rate of 41.2 % in all the cohort was used as the reference group for OR calculations

## Discussion

This study reported the efficacy of MII oocyte yield as reflected by cumulative live birth rates over a 6-month period in relatively young women with normal ovarian reserve. Each participant had only one oocyte retrieval after the same minimal ovarian stimulation protocol followed by single blastocyst transfer in a subsequent frozen embryo transfer cycle. Cumulative live birth rates were not affected by MII oocyte yield (Table 2). Interestingly, The number of MII oocytes had poor sensitivity and specificity for predicting a live birth.

In this study, all the participants had mIVF combined with single blastocyst transfer, which has the potential to reduce the risk of OHSS and the risk of multiple pregnancies without significantly lowering live birth rates [15]. This mIVF protocol used oral clomiphene citrate, thus allowing an endogenous FSH rise to be additive to the exogenous gonadotropins used for ovarian stimulation [16, 17]. Additionally in order to trigger oocyte maturation, this mIVF protocol used nasal (which is a patient-friendly route) GnRH agonist instead of the injectable hCG. Finally, the mIVF used a freeze-all embryo strategy in order to prevent any potential adverse impact of ovarian stimulation on the endometrial receptivity [18]. According to the Centers for Disease Control and Prevention (CDC) data on ART success rates of all American fertility clinics from 1997 to 2011 (http://www.cdc.gov/art/pubs.htm), success rates of both fresh and frozen-thawed embryo transfer cycles have increased over the last decade for women of all ages although the increase in success rates was greater in frozen-thawed embryo cycles (http://www.cdc.gov/art/pubs.htm). Another advantage of the freeze-all embryo strategy is that recent data showed that children born from frozen-thawed embryo cycles showed fewer perinatal morbidity and mortality compared to children born from fresh embryo cycles [19–21].

Increasing oocyte utilization rate will decrease the unnecessary production and retrieval of a large number of oocytes. Gonadotropins are administered to allow more follicles, which are already driven into the pool of developing follicles by gonadotropin-independent mechanisms, to escape follicular atresia. A challenging problem is to know the exact needed doses of gonadotropins and the extent of oocyte utilization rate since it is clear that not every retrieved oocyte is mature and gets fertilized to form a good quality embryo that end up being transferred or cryopreserved. In our study, the oocyte yield as categorized by low, intermediate or high did not affect the cumulative live birth rates. Thus it is reasonable to assume that gentle ovarian stimulation can produce reasonable pregnancy rates in patients similar to our participants, i.e., relatively young women with normal ovarian reserve. Another issue to consider is whether the number of retrieved oocytes influences the reproductive potential of the individual oocyte. Indeed, a study showed that although the pregnancy rate per cycle increased with the number of oocytes retrieved, the proportion of oocytes that produced good quality embryos dropped [22]. The authors of that study suggested that the production of too many oocytes could adversely impact the quality of the formed embryos.

Our results are consistent with other reports pertaining to fresh embryo transfer [11, 23] that assessed the efficiency of oocyte utilization in patients aged less than 38 years according to their oocyte yield: “low,” “intermediate,” or “high.” Similar to ours, their results showed that live birth rates were equivalent in the “low,” “intermediate,” and “high” oocyte yield groups. They also reported an apparent difference in the number of oocytes required for each live birth. In that study, “low” yield patients utilized 9.6 oocytes for each live birth, compared with 25.1 and 51.5 oocytes in the “intermediate” and “high” oocyte yield patients, respectively. These data taken together further support the hypothesis of oocyte efficiency in cases where there is a high oocyte yield since high oocyte numbers in our patients did not significantly increase delivery rates. One possible explanation for the inverse relationship between oocyte efficiency and ovarian response could be the negative impact of the supraphysiological estrogen levels on the embryo and/or endometrium [22, 24–26].

There are several limitations to our study. All patients received clomiphene citrate as part of their protocol, which can potentially cause a thin endometrial lining, thus they all had a freeze-all embryo strategy. Another limitation is the fact that we did not use serum anti-Mullerian hormone or antral follicle count to exclude women with diminished ovarian reserve, rather we relied only on age and day 3 FSH. Although we did not have the semen parameters on the male partners, the rate of ICSI use was not different among the three subgroups.

## Conclusion

In conclusion, this study extends the body of literature reported on conventional IVF to include similar data on minimal/mild ovarian stimulation IVF. Producing a low number of MII oocytes demonstrated a higher oocyte utilization rate without affecting live birth rates over a 6-month period using a single embryo transfer in minimal/mild ovarian stimulation IVF. Collection of large cohorts of oocytes does not necessarily benefit the patient. This information is useful in counseling patients undergoing IVF.

## Abbreviations

AUC: Area under curve
BMI: Body mass index
CI: Confidence interval
EG: Ethylene glycol
ES: Equilibration solution
FSH: Follicle stimulating hormone
GnRH: Gonadotropin releasing hormone
IVF: In vitro fertilization
LBR: Live birth rate
MII: Metaphase II
mIVF: Minimal/Mild stimulation IVF
OHSS: Ovarian hyperstimulation syndrome
OR: Odds ratio
ROC: Receiver operating characteristic
VS: Vitrification solution
